# Everyday practices at the medical ward: a 16-month ethnographic field study

**DOI:** 10.1186/1472-6963-12-184

**Published:** 2012-07-02

**Authors:** Axel Wolf, Inger Ekman, Lisen Dellenborg

**Affiliations:** 1 Institute of Health and Care Sciences, Sahlgrenska Academy, University of Gothenburg, Gothenburg, Sweden; 2 Vårdalinstitutet – The Swedish Institute for Health Science, Lund, Sweden; 3 Centre for Person-Centred Care (GPCC), University of Gothenburg, Gothenburg, Sweden

**Keywords:** Person-centered care, Moral stress, Interprofessional relationship, Professional-patient relations, Care environment, Care continuity.

## Abstract

**Background:**

Modern hospital care should ostensibly be multi-professional and person-centred, yet it still seems to be driven primarily by a hegemonic, positivistic, biomedical agenda. This study aimed to describe the everyday practices of professionals and patients in a coronary care unit, and analyse how the routines, structures and physical design of the care environment influenced their actions and relationships.

**Methods:**

Ethnographic fieldwork was conducted over a 16-month period (between 2009 and 2011) by two researchers working in parallel in a Swedish coronary care unit. Observations, informal talks and formal interviews took place with registered nurses, assistant nurses, physicians and patients in the coronary care unit. The formal interviews were conducted with six registered nurses (five female, one male) including the chief nurse manager, three assistant nurses (all female), two cardiologists and three patients (one female, two male).

**Results:**

We identified the structures that either promoted or counteracted the various actions and relationships of patients and healthcare professionals. The care environment, with its minimalistic design, strong focus on routines and modest capacity for dialogue, restricted the choices available to both patients and healthcare professionals. This resulted in feelings of guilt, predominantly on the part of the registered nurses.

**Conclusions:**

The care environment restricted the choices available to both patients and healthcare professionals. This may result in increased moral stress among those in multi-professional teams who work in the grey area between biomedical and person-centred care.

## Background

In the contemporary care environment, the need for strong person orientation rather than disease-centeredness is considered both important and self-evident. However, healthcare professionals still tend to focus on the disease within the person rather than the person with the disease [1]. Ethnographic studies suggest that biomedical knowledge takes precedence in multi-professional dialogue, and that even when physicians are not present, other professionals such as nurses act as ‘deputies for medicine’ [2]. It appears that the shift from traditional, disease-centred care towards more person-centred, multi-professional care is complicated and slow, and that a variety of challenges such as care fragmentation, time restraints and performance/target-driven organisations are slowing this shift [3,4].

Michel Foucault argues that modern healthcare and its organisation are based on the historical ideal of the medical discourse as a positivistic tradition built around objectivity [5]. In line with this thought, modern healthcare has adopted a routine-based method of organising care, reducing a unique, living person to a two-dimensional text entry in a lab test form or medical journal [6]. Patients are seen as generalizable, pathological processes rather than individual people in a particular context [7-9], and little scope is available for more subjective interpretations, *e.g.* a hermeneutical approach [10] or person-centred care (PCC).

The patient-healthcare professional interaction of today is somewhat standardised, regardless of whether it involves inserting a catheter or listening to the patient’s story [11].

This approach tends to transform the patient into a source of information rather than a partner in person-centred care [1]. Such a narrow, biomedical perspective may generate feelings of invisibility and inferiority in patients [12-14], and cause healthcare professionals to objectify patients [15]. PCC highlights the importance of both knowing the unique person that is the patient [16], and engaging with the patient as an active partner in the therapeutic alliance, sharing both power and responsibility [17]. The modern healthcare agenda is increasingly emphasising the importance of partnership between patients and healthcare professionals [17-20].

According to Long el al., understanding the hospital context is becoming increasingly important [21]. However, most clinical studies have only been concerned with specific practices or a certain profession [22]. These studies reduce the clinical setting to a series of isolated interactions or professions, commonly disregarding the complexity of the care environment [23]. The modern clinic is a social world comprising patients, nurses, physicians and other care providers who work at the clinic [10]; as well as a physical context [24-26]. By reducing the clinical world to a series of isolated situations, several studies investigating PCC have targeted specific areas of care, or the patient-physician/patient-nurse relationships [17,27,28]. The dynamics of a clinical setting and its resistance to transition also need to be understood in relation to the complex social world in which the setting exists [29].

Few ethnographic studies have targeted the everyday spectrum of relationships between different kinds of professionals and the patient within the hospital setting [21]. We lack knowledge of how the routines and values of the care environment influence patients and healthcare professionals in everyday hospital practice [22,30]. We aimed to describe the everyday practices of professionals and patients at an internal medical ward in order to analyse how the specific care environment, with its routines, structures and physical environment, influences the purposes, actions and relationships of both patients and healthcare professionals.

## Methods

### Study design and study site

This study is based on data collected during a 16-month fieldwork period (between 2009 and 2011) in a Swedish coronary care unit. One male and one female researcher (the first and third authors) conducted the fieldwork. The main methods used to collect data were participant observation, informal talks and formal interviews. During the fieldwork, the discrepancy between what people say and what they do was explored. This is invaluable in the study of complex, multi-professional settings such as a hospital clinic. Ethnography involves ‘thick’ descriptions [31] of social phenomena focusing on a participant’s way of understanding a situation [10]. Besides acting as a set of techniques for observing social phenomena, ethnography is a particular way of seeing. It is a reflective process in which the researcher strives to see beyond what is taken for granted by themselves and the participants [32].

The fieldwork was carried out in a coronary care unit at a Swedish university hospital with a conceivable catchment area of over 250,000 residents. The ward comprises an intensive care unit (six single rooms) and a step-down/rehabilitation unit (13 rooms). The unit has 36 beds in total. Each sub-unit has two teams during the day shift, consisting of a registered nurse (RN) and an assistant nurse (AN) caring for 4–5 patients, and both teams shared one senior cardiologist and one resident. During the evening, night and weekend shifts, each team was responsible for 10–15 patients and an on-call physician served the whole ward. The most common diagnoses during 2009 were atrial fibrillation, myocardial infarction and chest pain. The average length of stay was 4.4 days. The staff consisted of 60 RNs, 40 ANs and 35 physicians. Of the 35 physicians, 14 were senior cardiologists and two were professors. About 40% of the physicians and 75% of the nurses were women. The vast majority of the ANs were women. The professionals and the administrative management staff were open-minded and curious about the study and welcomed the researchers and their team onto the ward.

### Fieldwork and participants

Participants were observed over their entire work shift (6–10 hours) during the day, evening and night shifts. Observations were made once or twice a week, and in periods during a whole working week. Working separately, the researchers followed the staff during their everyday activities, and helped them with some basic nursing duties such as food distribution and assisting patients getting in and out of their beds. While the quality of an ethnographic study should not be measured by length of its fieldwork, a minimum ethnographic fieldwork length of at least 12 months is considered desirable [32].

The researchers wore hospital uniforms and nametags with the wording “researcher”. In situations such as examinations or invasive procedures the researchers did not actively participate, but observed the situation. The researchers attended social gatherings such as parties and planning days in order to get to know the professionals in settings outside the ward. Field notes were taken during the observations and informal talks. At the end of the day, the researchers used the notes to write detailed descriptions of the field experiences. The analysis began at the point when the ethnographer reflected on the observations and conversations in relation to earlier experiences in the field. The aim of the observations was to gain an understanding of the complex everyday aspects of the ward, as well as the relationships between professionals and between professionals and patients. Formal semi-structured interviews, in which only a few questions were prepared so as to follow the interviewee’s line of thought, were performed with six RNs (five female, one male) including the chief nurse manager, three ANs (all female), two male cardiologists and three patients (one female, two male). The participants were strategically selected according to their occupational group, sex, and age. All formal interviews were digitally recorded and transcribed verbatim.

### Data analysis

The care environment comprises the physical setting, organisational systems, professional competencies, human relationships and hierarchies [33]. Innovation, risk-taking and decision-making take place in the context of the care environment [33]. In our analysis, we were influenced by the philosopher Kenneth Burke’s dramatism method [34]. This method aims to generate principles for understanding human motives by describing the constant interaction that occurs between the environment and its agents, and analysing how this interaction influences what people do and why they do it. Central to the dramatism method are five terms, which Burke calls the Pentad: Scene, Act, Agent, Agency and Purpose. We found the Pentad useful for structuring our material and analysing the complex clinical setting.

· The Scene is the specific location of the ward, which according to Burke comprises the physical setting as well as the cultural and historical structures.

· The Act denotes what the professionals and patients actually do during an ordinary day on the ward.

· The Agent is the one who performs the act, *e.g.* a patient, physician, AN or RN.

· The purpose refers to the agent’s intentions.

· The agency illustrates how the agent acts to fulfil the purpose, *i.e.* the means or instruments they use.

Burke suggests that acts and purposes are shaped by the specific scene, which greatly influences agency, and that the scene is the context in relation to which a person’s agency needs be understood. In our study, the care environment is the context in relation to which the actions of the patient or healthcare professional need to be understood [33].

The method used for interpreting the field notes and interview texts was influenced by Paul Ricoeur’s hermeneutic idea of a circular movement between ‘understanding’ and ‘explaining’ the text [35,36]. As with the actual experience of the researcher, their preunderstandings are also part of the knowledge that is being created. The researchers’ preunderstanding of the clinical context differed in important ways. The first and second authors are both RNs with clinical experience of care and the function and roles of healthcare professionals. The third author is an anthropologist, experienced in field studies both abroad and in Sweden, but with no medical or nursing background. The authors’ divergent preunderstandings were fruitful in the interpretation process as they saw different things in the material. The authors discussed the material extensively in order to triangulate their observations and interpretations.

### Ethics

Permission to conduct the study was obtained from the Regional Ethical Review Board in Gothenburg, Sweden, and the investigation conforms to the principles outlined in the Declaration of Helsinki. The staff received written and verbal information about the study at various staff meetings, and were asked for their consent to be interviewed. Every observation session was preannounced at the start of every shift, and the chief nurse manager or other staff member assigned the researcher to one of the nursing teams. If one or more of the team members expressed discomfort with the current observation, the researcher switched to a new team. If a patient expressed discomfort either directly or by way of the nurses, the researcher switched to another team. During the entire fieldwork period there were very few occasions when staff members or patients expressed discomfort with the observation. However, being aware of the ethical sensitivity with observational studies in a hospital ward, the researchers switched teams at the slightest apprehension that a staff member or patient might feel unease with the researcher’s presence. The observations did not seem to influence those observed, as they were used to having a steady stream of students and researchers on the ward. Sometimes the staff expressed curiosity or discomfort about the field notes, which we responded to by taking the opportunity to discuss them with those involved. Written consent was obtained from the patients prior to the formal interviews and verbal consent was obtained during encounters. As noted, we were aware that patients and staff might find it difficult to say no to an interview or observation, so we were sensitive to non-verbal signs of discomfort with the situation.

## Results

### The scene

The ward had a rapidly shifting agenda with high patient admission and discharge rates, daily staff rotation and high replacement rates of different care professionals and students. Both nurses and students placed great emphasis on acquiring biomedical knowledge and mastering pharmacology and technical skills such as electrocardiogram (ECG) interpretation and auscultation using a stethoscope. They considered these areas of knowledge vital in order to be recognised as competent.

From an outsider’s perspective, the ward is designed for surveillance and primed for emergency, lifesaving interventions. Consisting of eight offices for nurses and physicians, the ward is in the shape of an ‘H’ with the rooms running along two long corridors, painted in bright yellow, linked by a shorter corridor. The entrance is at one end and the patients’ dining hall at the other. ECG machines and defibrillators stand along the corridor walls, constantly available. Surveillance monitors on the walls with various signals and alarms are a constant reminder of the close relationship between life and death on the ward, relentlessly reminding the personnel to be prepared for an emergency. From a patient’s perspective, the ward actively works against their need for normal human activities. There are few places to meet family and friends or to chat with other patients. Everyday activities, such as reading the newspaper or watching the news are difficult because the common room (with the television and kitchen) is often used for staff meetings. The smaller television room is frequently used by staff or turned into an extra patient room at times of overcrowding. Patients are told to stroll in the corridors or sit on their beds because the patient rooms have very few chairs. Visitors are welcome during visiting hours (11 am to 7 pm). No family members are allowed to stay overnight, except if the patient is staying in one of the few single rooms. The reason given is that in an emergency, such as a cardiac arrest, the staff must be able to act quickly and there must be no obstacles to carrying out the emergency care procedure. Both patients and care providers had to accept these conditions of the care environment.

Another condition in the care environment was the routines. The care activities on the ward revolved around three periods: morning, evening and night. The mornings were the productive time for treatment and care decisions, while the evening, night and weekend shifts were focused on surveillance. The central activity in the morning was the medical rounds. The rounds were divided into two sessions held in the physician’s office in each sub-unit. The first and longest session was closed to the patient. This was when all of the professionals (RNs, ANs and physicians) met to plan each patient’s care. For reasons expressed by the staff as safeguarding the patients’ confidentiality, patients were referred to in terms of room and bed number, such as 11:1, meaning room 11, bed 1 when talking in the corridors or public spaces in the ward where their conversations may be overheard by relatives or visitors. This referral to patients in terms of bed number was also observed during the medical rounds held in the physician’s office. When meeting the patient in person, the staff used the patient’s first name. During the second part of the rounds, patients were visited at their bedside. The physicians spent more time during the rounds reading the patients’ records than talking to the patients. The experienced physicians said that it had not always been like that and they complained about the new patient record system, which was time-consuming. A male cardiologist also stated that even though he could not always obtain much information on the latest developments in the patient’s condition from the nurses, he could acquire a fairly good picture as he talked to the patient. He pointed out that the patients’ own accounts did not always concur with what was documented, which is why he preferred to start from the beginning: *“If it’s a patient I’ve not met previously, I usually ask about what has happened; I carry out a new inquiry, a new anamnesis. It can be quite different from what I’ve read earlier…”*

Generally, the patient has a few minutes with the physician, sometimes less, sometimes more depending on the amount of information and the level of inquiry. Both ANs and RNs have individual working schedules, which results in high staff circulation, so patients meet numerous physicians and nurses during their hospital stay. The RNs and ANs find this schedule a great help with their private lives, but also recognise its disadvantages. Starting again with new patients, sometimes several times a week, was clearly a stress factor that had a negative impact on both patient care and professional teamwork, and resulted in a discontinuity in care provision.

### Everyday practices (agents, purposes and agency)

For a summary of the agents and their everyday acts, see Table 1.

**Table 1:** Summary of the selected agents’ everyday acts on the ward

**The Agent**	**The Act**
The patient	The patients were mostly sitting or lying on their bed, waiting for a decision concerning their treatment. Generally, they preferred not to disturb the nurses by ringing the bell or posing questions if they were not in acute need.
The assistant nurse (AN)	ANs supported the patients in their daily needs. They spent most of their time with the patients and reported to the responsible RN. The ANs seldom stayed a whole week in the same subunit.
The registered nurse (RN)	RNs communicated with patients, their family and all care providers inside and outside the hospital who were involved with the patient. The RN had a central administrative responsibility and coordinated the administrative, logistical (lab, examinations, transport) and medical inquiries. The RNs seldom stayed a whole week in the same subunit.
The physician/resident	The physician was stationed at the ward for a week, and two days per week they were in charge of the subunits’ patients, backed up by the senior cardiologist who was usually absent from the ward on those days. Otherwise, the physician assisted the cardiologist with mostly administrative tasks such as writing referrals and discharge notes, reading the journals in the morning and reporting to the senior cardiologist in case they had not had time to prepare themselves. The physician also had specialist out-patient clinic duties and circulated between different wards and hospitals as part of their training. The physician stayed in the same subunit for one week at a time.
The cardiologist	The cardiologist had the overall responsibility for the patients in their subunit during the week. They held the medical rounds in the ward three days per week. Their tasks included journal documentation, preparation of discharge notes, answering referrals and on-call duty in the ward and the entire medical clinic during evening and nights. Besides these tasks, the senior cardiologist had many obligations outside the ward, such as consultations, research assignments, and supervision of residents and medical students. The cardiologist mainly stayed in the same subunit for one week at a time.

### Patients’ purposes and agency

The patients’ main purposes were to understand what had happened to them, why they had been hospitalised, what would happen next and how the disease and the treatment would affect them and their future. Observations revealed how difficult this was and how they developed various strategies to achieve these purposes. Many patients described their hospital stay as tedious and filled with waiting for answers and information about their care and treatment. An effective strategy for patients to keep themselves informed about their situation and to receive good care was to build up a good relationship with the care provider, mainly by being cheerful and showing gratitude, accepting the routines and following the care providers’ instructions.

The morning round was seen as the epicentre of the day, when the patients were able to meet with the physician who might be able to provide some information about their care plan. When they met the physician, patients often felt they had to answer the same questions posed by previous physicians: *“And so I repeat my story again”* an elderly male patient said with a smile. Furthermore, patients frequently became stressed by the large number of people accompanying the physician, by the fact that other patients may overhear the conversation, and by the stressful atmosphere: *“They [the physicians] are always in such a hurry”* was a common comment. An older male patient said that one strategy was to be alert: *“If you want to pose questions you need to be quick [as they are soon on their way to the next patient].”*

After the rounds, others ask the nurses about what the physician had actually said and about the things that the physician had not said that still concerned the patient: *“The ones you can talk to are the nurses, they will tell you in plain Swedish what is going on and they have time [for you]”* stated a male patient in his 60s. However, for information about their treatment and condition, another man stated that nurses were usually reluctant to answer such questions and referred the patient to the physician.

Noticeably, patients often expressed awareness of the care providers’ workload, taking responsibility for the working situation by showing a reluctance to “disturb” the care providers. The patients also expressed the importance of being nice and pleasant, as expressed by a male patient in his 60s: *“It depends on the patient too. ‘You are always happy in the morning’, they say [referring to the staff]. Sometimes I’m not, but I give them a smile anyway. It has such great importance… those small things….”* Sometimes, however, the importance of being pleasant could become rather arduous. A female patient who was liked by the professionals because of her smooth manner and was otherwise very content with her time on the ward, emphasised a concern with a felt pressure to be nice when she said: *“We’re here because we’re sick, and it is important that the personnel remember that.”*

Although both the RNs and ANs encouraged patients to contact them, many said they would not ring the bell or ask questions unless it was in an emergency. Patients tried to adapt to the pressurised environment and find alternative sources of information by searching the internet, having discussions with other patients or trying to read the surveillance monitor on their own. Patients commonly observed the monitors, trying to figure out the ECG curves and asking the nurse: *“how do I feel*?” In an interview with a male patient, this common distancing of the patients from their own body was expressed as *“the oxygen saturation is low, and needs to be higher before I can go home*.*”*

### The ANs’ purposes and agency

The ANs aimed to be seen as a competent, professional and caring part of the multi-professional team who supported the patients’ wellbeing and satisfied their everyday needs during their hospital stay. The ANs’ collective understanding was that they were the ones ‘who know the patient,’ *i.e.* the ones in the team who spent the most time with the patients. A female AN stated in an interview: *“We are with the patients, giving them clean clothes in the morning, and even if there are RNs that help patients too, we are the ones who are actually there for the patients all day, helping them with all the necessary things, such as brushing their teeth, serving food and so on.”* The ANs were proud of this task and their work. However, some of them expressed feelings of not being appreciated for their skill. A change in the routines that the ANs welcomed was that they became expected to assist in the morning rounds. A female AN stated that her presence during the medical rounds had improved her perception of herself as a ‘professional’: “*it gives me a better picture of the patient’s overall situation.”* She explained that she felt she was in a better position to answer patients’ questions about their situation, which made her feel more capable in her dealings with them.

Observations revealed that ANs were relatively silent during the rounds. Normally, they only answered direct questions from the physician or RN with short comments. These were usually in relation to discussions between the nurse and the physician concerning the patient’s nursing status and general state of wellbeing. ANs expected to join the rounds, and most ANs did; however, their presence was not mandatory and no one seemed to enquire if an AN did not show up for the rounds. ANs could simply tell the responsible RN that they were going to skip the rounds. Reasons for not joining were usually related to their workload: *“If the patients are in need, they naturally take priority over the rounds.”* ANs could also skip the rounds if they felt it was a waste of time, if they felt uneasy with the rounds situation or if they felt it would be pointless following the rounds since they were only scheduled to work at the sub-unit for one day.

### The RNs’ purposes and agency

The RNs’ aims were to provide good nursing care, to learn how to manage and master emergencies and to expertly perform the physicians’ care programme. Observations revealed that in this particular care environment the RNs’ multiple aims tended to be juxtaposed with the care environment. The established metaphor of the RN as ‘the spider in the web’ highlights their role in the team. They co-ordinate all care providers involved with a patient. ‘The spider stretched in the web’ might be an even more appropriate metaphor, as the nurses frequently felt torn between the various needs of the other care providers, the patients and their relatives.

The high value assigned to biomedicine and the skills for managing serious diseases in this particular care environment were evident. RNs felt that they needed to be well informed about vital signs and the patient’s medical problems in order to be acknowledged as competent by their colleagues, especially other RNs: “*Some colleagues interrogate you during the oral patient shift report, sometimes they even question why a specific treatment was initiated or an examination was not performed,*” a female RN stated in an interview.

To acquire a comprehensive picture of the patient that could be passed on to the physician during the morning rounds, the RNs usually focussed on medical information and the patient’s vital signs as derived from the patient’s records. They said that they did not have time to meet and really talk with the patients while also reading and memorising data from their records. The conversations during the rounds were mainly related to medical questions and treatment. RNs, like ANs, usually responded to the physicians’ direct questions concerning the patients’ well-being and response to treatment, normally supported by the ANs’ comments and observations. RNs described the knowledge related to their discipline and profession as difficult to transfer to reports and clinical practice. An RN expressed in an interview that *“writing a memo about a UCG [Echocardiography] examination is easy, but it’s harder to write down the patient’s feelings, anxiety and support needs on a piece of paper [in the journal].”* The nursing perspective was given relatively little attention during the medical rounds, and was thereby effectively de-prioritised. The RNs often felt uneasy and guilty about this reduced nursing perspective and about not seeing the patients enough to adopt this perspective. One young male RN said *“I only have about five minutes with every patient during my shift, so I have to make the best of it. I read the nurses’ and physicians’ documentation in the patients’ records to get the whole picture.”*

Because of the rotating schedule, the nurses often found themselves with a whole set of new patients. The RNs seldom stayed a whole week in the same subunit. The feeling of always being one step behind was overwhelming. Overall, the explanation given by the RNs for the vague nursing perspective was the lack of time. The RNs felt that they were drowning in administrative work and spent more time on the phone and in front of the computer than with the patients. In conversations, the RNs sometimes reflected on, as they put it, the absurdity of routines taking precedence over the patients’ needs. An illustrative example was an episode that occurred when a patient died. The days before, a breakout of gastric flu had seriously diminished the workforce on the ward and the staff seemed exhausted. Two RNs were frustrated and disappointed about not having been able to provide good care to a patient who was terminally ill and died during the morning. In the commotion, the nurses had proceeded with the morning routines without asking the ward manager for more nursing staff in order to focus more resources on this particular patient. The two nurses tried to console each other by saying that it had been stressful to administer medicine to all of the patients, prepare for the medical rounds and on top of that try to provide this terminally ill patient with good nursing care. They felt that they had let down both the patient and his relatives. One of them said in a later conversation with one of the researchers: *“I have experienced dying patients previously, but not being able to give good nursing care or a single room… What kind of care is that?”*

The RNs sometimes transferred to less stressful units citing reasons of stress and the high level of medical competence required to care for patients who were often very ill. However, the majority of the nurses expressed satisfaction with the care environment, with its air of emergency and high circulation of patients and staff. Although stressful, it presented a variety that they found challenging in a positive way. *“You never know how your working day will proceed”* and *“You always learn something new”* were common comments.

### The physicians’ purposes and agency

The purpose of the physician was to know and understand the patient in order to decide on and co-ordinate suitable care and treatment. For the physicians, to know and understand the patient typically implied knowing the patient’s pathology and medical state. According to a male senior cardiologist, achieving this goal *“is all about how you succeed in obtaining information about the patient,”* adding that the rounds were his main working tool. For him, seeing a patient for a few minutes is often sufficient to put together a picture of the patient’s state of health. The same cardiologist stated *“the aim is to get the right information from the patient in the shortest period of time.”* Another male cardiologist emphasised that sometimes it is sufficient to simply watch the patient from a distance. According to the physicians, the ideal round was one where they had met the patients before, and more importantly, where the RN knew them and their history. However, because of the individual working schedule, which the physician said did not prioritise continuity with the patients, nurses have frequently not met the patients before. Further complicating the potential to get to know the patients, the physicians also circulated between the subunits on the ward and had a whole set of ten new patients every Monday. Many physicians pointed out the absurdity of this and the resulting prolonged length of stay for each patient. A senior cardiologist stated *“It becomes ineffective care… You will lose pace by changing staff all the time; length of stay will surely be prolonged. You lose two or three days as soon as you alter the cardiologist.”*

Furthermore, physicians frequently had to change their schedule at short notice because management ordered them to cover for an absent colleague. Sometimes this meant combining their tasks and those of their colleagues. *“We physicians have exceptionally many different tasks to perform, our workload has accelerated the last couple of years,”* a cardiologist said talking about the numerous responsibilities the physicians, in particular the senior cardiologists, had at the clinic. The senior cardiologists were heavily involved in consulting within and outside the clinic, and were occupied with research and specialist outpatient clinics. They were constantly on the move and spent a great deal of time on the phone. Other colleagues and RNs demanded that the physicians were available on the phone at all times for consultations, which is why they usually answered calls even during lunchtime. The senior cardiologists often emphasised that *“working as a cardiologist, you must like and be able to work under great pressure.”* It was perceived as being part of the allure of the job. For the residents, the time on the ward and the specialist outpatient clinic were part of their training and one of several stations along their career path. Like RNs, they typically felt squeezed for time and ‘stretched in the web,’ having to respond to the needs of patients and their relatives as well as nurses and cardiologists. Residents seemed to regard this arduous time as a step in their career rather than an infinite situation, which made it easier to bear.

## Discussion

In this study, we explored a particular care environment in a coronary care ward. With its architecture and cultural characteristics, the care environment effectively reduced the freedom of both patients and healthcare professionals to work towards achieving their different goals. Differentially suiting the various healthcare professionals’ purposes, the prerequisites of the care environment suppressed multi-professionalism and PCC by creating demarcated routines rather than holistic care. These routines, often performed to tight deadlines and in a ritualistic manner, give little opportunity for the care providers to reflect on their actions and the choices they made. In particular, the RNs reacted to the restriction of choice with feelings of guilt and inadequacy. They felt trapped in an ambivalent state between a desire for status achieved through biomedical knowledge, technical skill and adherence to routines, and their professional perception of good care, which included marginalised nursing perspectives.

### The care environment restricted person-centred processes

According to Burke, the different parts of the Pentad (scene, act, agent, agency and purpose) interact and influence each other [34]. Important for our argument is that Burke’s Pentad demonstrates how acts are created or obstructed by a specific scene. The scene establishes the purpose and is the context in relation to which different forms of agency are created. Burke’s Pentad is useful for understanding and explaining the relationship and interaction between the parts and the whole of the complex clinical social world. The physical setting and the architecture were prominent elements of daily care, and set the scene for all those involved, both healthcare professionals and patients. In our case, the ward environment served to maintain a medical structure by promoting clinical, time-bound, routine-based care founded on objective signs and guidelines that offered few opportunities for subjective interpretation.

Patients not only adapt to their illness, but are also in constant interaction with their environment [37]. The care environment, with its prominent medical discourse, seriously restricted the patients’ potential to act as an independent person in everyday activities. Patients were often uncertain about what to expect and when to expect it. For example, they rarely knew when the morning medical rounds or consultations would take place because the rounds extended over several hours. This uncertainty meant that patients were ‘passive passengers’ in their beds, as they wanted to be available for the healthcare professionals when it was time. Most patients tried to establish a good relationship with the healthcare professionals by creating a reputation for being a good, obedient patient who followed the rules of the care environment.

In a recent study of patients admitted to a general medical ward in Australia, observations suggested that for over 88% of their stay patients were located in their rooms, being inactive and unaccompanied for the majority of their admission [38]. Previous studies have pointed out the importance of having an environment that promotes activity and social interaction [13,24], suggesting that the ability to participate in everyday life on the ward is important to the patients as a way of convincing themselves of their ability to manage at home after discharge [13]. However, as discussed by Long *et al.*, hospitals are liminal spaces [21], where the patients commonly lose their identity because every day routines and personal resources are turned into medical needs. The routines on the ward promoted patient passivity, and created an environment that suited the healthcare professionals’ agency and purposes more than those of the patients.

Foucault compared the institutional architecture of a hospital ward with other institutions, such as a prison [39], and suggested that like a prison, a hospital is built for observation of the object (the patient) and is designed and constructed to suit those who observe (the healthcare providers). Similar to Foucault’s thoughts, our findings suggested that to “know the patient” seemed to equate to knowing the “body pathology”. The surveillance monitors, radiological examinations and blood samples formed the panoptic observation tower of the care environment, ingeniously designed and constructed to suit those who observed, *i.e.* the healthcare providers.

The study care environment was suited primarily to the physicians’ purpose of diagnosing and treating the disease. However, it seemed as if the care environment was suboptimal even for the physicians. They were often dependent on the RNs’, and in some cases the ANs’, observations and interpretations of the patient in order to make decisions, and were frustrated at the lack of information concerning the patient’s psychosocial state or similar. The information they received from the RN was basically biomedical in nature and the RNs reported events that the physicians could read about in the patient records.

According to Lock and Gordon [40], biomedicine as a science is socially constructed, making the high status of biomedicine a tool of social control. The recent rise of concepts such as “shared decision-making” and “patient empowerment” suggest an effort by governments and health organizations towards increased autonomy and self-care responsibility of the individual patient [41]. The question is how the traditional stronghold of biomedical science, *i.e.* the hospital structures, will respond to this uprising and change in the political agenda. These cultural structures are often subconsciously maintained and passed on by the healthcare professionals [42]. Physicians, and especially RNs, are educated to provide care that includes a biopsychosocial dimension of the patient [7,43]. Yet the cultural characteristics of the ward, such as the language, technology, clothing and symbols [44,45], were so dominated by structures that objectified the patient that the highly trained professionals had difficulty maintaining the patient perspective.

One example of this patient objectification is the referral to the patient in terms of a bed number instead of their name. While the staff claimed reasons of confidentiality in public areas of the ward, the patients were also referred to as a bed numbers during the medical round that was closed to visitors. This dehumanizing and objectifying way to talk about the patients remained even in situations where confidentiality was not threatened. Furthermore, because of the high daily nurse circulation, referral to patients as bed numbers rather than their names could be seen as an act of necessity rather than confidentiality.

While there was an almost daily interpersonal as well as temporal discontinuity between the nurses (RN and AN) and the patients, the physicians were usually responsible for the same patients for a week. Whereas there are several studies on the physician-patient continuity of care and its outcomes [46,47], to our knowledge, there are few studies on the general nurse-patient continuity in hospital settings and its impact on satisfaction (of both patient and nurses) and clinical outcomes. Studies suggest that less than 50% of staff time is spent with patients [48], and that RNs spend less than 40% of their time with patients [49]. Studies also demonstrate that individual patients spend less than approximately 13% of their length of stay with nurses (11%) or physicians (2%) [38]. In our study, the organisational structure whereby nurses had an almost daily turnover of patients might have increased the nurses’ feeling of always being “one step behind”. While care continuity may improve patient satisfaction, our results illuminate the need for future research about care continuity and nurses’ moral stress levels.

The nursing perspective was marginalised in the care environment. While both the physicians and RNs acknowledged that the ANs were closest to the patients, neither of them gave the ANs much time to share their knowledge of the patient. The RNs, in contrast to the ANs and physicians, expressed feelings of being torn between ideals and reality, *i.e.* between the theories of nursing and the structures and daily practices of the care environment. In the example of the terminally ill patient who died during the morning routines, the nurses tried to squeeze in care of the dying patient with all of the other routines and the medical rounds. Although excessively burdened with tasks and routines, no request was made for more staff resources. They struggled with their conscience, knowing that the inevitable event of death seldom gives way to the routines and demands of the care environment. Because of the stress and importance attached to routines, two RNs prioritised morning routines over palliative care.

Our findings suggest that the care environment gave different individuals fundamentally different prerequisites for fulfilling their purposes, and left little room to make choices or interpretation outside the set routines and structures. This is an important finding, suggesting that future changes in different healthcare systems, such as striving towards more PCC, must consider the complex interplay within the care environment; including the physical design, cultural characteristics and the multi-professional teams’ different prerequisites for fulfilling their purposes.

### Moral stress/consciousness

In the present study, all those involved made active choices. For example, the patients chose passivity and obedience in order to fulfil their purposes within the constraints of the care environment. Another finding was that the RNs, in contrast to the ANs and the physicians, reacted to the perceived scarcity of time and inability to fulfil their function with feelings of guilt. On a regular basis, the RNs had to make choices between the care environment’s promotion of medical discourse and their nursing ideals. It was the RNs, not the physicians or the ANs, who were expected to continuously respond to the patients and deal with their worries and thoughts about their situation and future. Thus, the RNs were in the best position to see the lack of participation and dialogue offered to the patients within the care environment.

It has been suggested that the conflict between the values of nursing care and the realities of the care environment can cause RNs to suffer such feelings of inadequacy and guilt that they even avoid patients [50]. In the present study, we observed that the RNs were clearly sandwiched between different demands, routines and timeframes, which created feelings of inadequacy. Moral stress [51], or the closely connected concept of stress of conscience [52], surfaces when the care environment and organisational aspects present values that conflict with the nurses’ or healthcare providers’ moral convictions [53]. Specifically, moral stress surfaces when external factors prevent healthcare providers from doing what they believe is best for the patient, or provide them with few choices regarding how to act in a certain situation [51].

When considering the failure to spend sufficient time with patients, most RNs expressed strong self-criticism. However, physicians seemed generally content with the time spent with patients. In cases where they were not content, they attributed this to structural problems on the ward rather than personal shortcomings. As suggested, the physicians’ purposes were basically consistent with the care environment, which in turn resulted in the physicians’ having more scope for agency, *i.e.* a greater spectrum of choices as to how to act in order to fulfil their purposes. This in turn may lead to a greater perception of control over the situation.

The ANs expressed few feelings of guilt or lack of control, and we observed that the ANs achieved their primary goal of attending to the patients. They expressed pride in their knowledge and skills even though they felt they were not really valued by the care environment. The RNs on the other hand expressed feelings of a lack of control, frustration, guilt and ambivalence. Our findings suggest that those who were at risk of moral stress were the RNs. Previous research has suggested a link between healthcare professionals’ stress of conscience and burnout [50,54]. Although most nurses on this particular cardiology ward explicitly stated that the atmosphere of emergency and stress thrilled them, moral stress was something that was expressed more implicitly in private conversations and was often linked to difficult care situations observed by the researchers.

### Feeding the care environment

Our study highlights the complex interplay between the structures that uphold routines, attitudes and practices and the potential for individuals to act according to their own agenda and moral beliefs. The anthropologist Sherry B. Ortner’s ‘serious games’ concept [55] theorises about the complex social realities in which individuals are neither ‘autonomous agents’ nor stuck in a structure without agency, implying that structures curtail but do not eliminate the possibility to make choices. Life is a game and people play according to the rules with wit and intelligence. Everyday decision-making processes in the emergency care setting are formed and nourished by the socially constructed games and hierarchy that occur primarily between the RN and physician [56]. As such, the games on the ward effectively diminish multi-professionalism and PCC.

Internationally, the importance, but also the complexity, of PCC is recognised [41,57]. In the present study, several research projects on the prerequisites and effects of PCC were on going within the hospital. PCC comprises both a mind-set of how the healthcare provider should acknowledge the patient as a partner in order to grasp the patient’s lived experience, *i.e.* person-centeredness [1], and a structured approach to clinical practice, *i.e.* PCC methods [58]. Ekman *et al.* previously showed that a structured PCC approach to patients suffering from chronic heart failure yielded a significant positive clinical effect if fully implemented during the entire admission period [58]. In their study, only 60% of the patients received continuous PCC during their entire length of stay. This suggests that PCC may be difficult to fully implement without continuous efforts to change the structures, as well as the actors’ (both staff and patients) incentives to act differently.

One important motivational strategy could be clinical education and time for reflection, achieved for example through a drama program [59,60]. Nevertheless, clinical education and reflection will not reach their potential if the care context remains the same. A desire to change the context needs to be shared throughout the entire organisation, from management to the staff on the clinic floor [33]. One intervention will not change the context, rather many small and different aspects and ideas will enable the context to change [57].

Cultures, like the culture on the study ward, are webs of significance that we ourselves have spun, *i.e.* they are lived and created by their members [31]. Although the care environment in this study seemed to have what is an almost iron grip on the patients and healthcare professionals, these parties nevertheless hold the key to changing the specific care environment.

### Limitations and strengths

The voices of the patients were less prominent in this article because our focus was on the healthcare professionals’ purposes and agencies in this particular care environment. The study was carried out in a specific medical ward, at a specific university hospital, in a large city in Sweden. Having only conducted fieldwork in one ward could be seen as a limitation for generalisability. However, the results were not intended to be representative of medical wards inside or outside of Sweden. The aim was to provide a deep understanding of the complex care environment, emphasising the importance of knowledge of the local care context in order to implement changes [33]. The extensive period of time that the two researchers with different backgrounds and preunderstandings spent on the ward strengthens the validity of this article.

## Conclusion

This study described a care environment that contains multi-professional competence yet promotes only ‘one truth’: the positivistic biomedical truth. The care environment differentially influenced the patients’ and healthcare professionals’ abilities to fulfil their different purposes, and produced feelings of guilt and inadequacy in the RNs, which may lead to moral stress and burnout. We suggest that if the healthcare system wants to adopt PCC, changes in the ward architecture and the multi-professional teams’ ability to fulfil their moral conviction, as well as patient education about what PCC means, will be necessary.

## Competing interests

The authors declare no conflict of interest with regard to the authorship and/or publication of this article.

## Authors´contributions

AW: Study design, data collection, data analysis, preparing the manuscript. LD: Study design, data collection, data analysis, preparing the manuscript. IE: Study design, preparing the final manuscript. All authors read and approved the final manuscript.

## Pre-publication history

The pre-publication history for this paper can be accessed here:

http://www.biomedcentral.com/1472-6963/12/184/prepub
